# Comparison of Different HILIC Stationary Phases in the Separation of Hemopexin and Immunoglobulin G Glycopeptides and Their Isomers

**DOI:** 10.3390/molecules25204655

**Published:** 2020-10-13

**Authors:** Katarina Molnarova, Petr Kozlík

**Affiliations:** Department of Analytical Chemistry, Faculty of Science, Charles University, Hlavova 8, 128-43 Prague, Czech Republic; katarina.molnarova@natur.cuni.cz

**Keywords:** glycoproteomics, glycopeptides, separation of glycopeptide isomers, glycopeptide separation, hydrophilic interaction liquid chromatography

## Abstract

Protein glycosylation analysis is challenging due to the structural variety of complex conjugates. However, chromatographically separating glycans attached to tryptic peptides enables their site-specific characterization. For this purpose, we have shown the importance of selecting a suitable hydrophilic interaction liquid chromatography (HILIC) stationary phase in the separation of glycopeptides and their isomers. Three different HILIC stationary phases, i.e., HALO^®^ penta-HILIC, Glycan ethylene bridged hybrid (BEH) Amide, and ZIC-HILIC, were compared in the separation of complex *N*-glycopeptides of hemopexin and Immunoglobulin G glycoproteins. The retention time increased with the polarity of the glycans attached to the same peptide backbone in all HILIC columns tested in this study, except for the ZIC-HILIC column when adding sialic acid to the glycan moiety, which caused electrostatic repulsion with the negatively charged sulfobetaine functional group, thereby decreasing retention. The HALO^®^ penta-HILIC column provided the best separation results, and the ZIC-HILIC column the worst. Moreover, we showed the potential of these HILIC columns for the isomeric separation of fucosylated and sialylated glycoforms. Therefore, HILIC is a useful tool for the comprehensive characterization of glycoproteins and their isomers.

## 1. Introduction

Glycosylation is one of the most important and extensive post-translation protein modifications [1]. Protein glycosylation includes a complex series of enzymatic steps that result in the formation of protein-bound oligosaccharides with various biological functions [1,2,3]. Alterations in glycosylation patterns affect many biological processes, such as protein folding, intracellular sorting, secretion, and host-microbial recognition [4,5]. In addition, abnormal protein glycosylation has been used as a diagnostic tool for many cancers, including breast, prostate, and ovarian cancer [6,7,8,9]. Yet, despite recent bioanalytical advances in glycomics, glycoprotein analysis remains a challenge.

The most commonly used method for glycoprotein analysis is reversed-phase liquid chromatography (RP-LC) with tandem mass spectrometry (MS/MS). However, RP-LC is unable to adequately resolve glycopeptides into their glycoforms [10,11,12]. An alternative chromatographic mode to RP-LC in glycopeptide analysis is to use porous graphitized carbon (PGC). PGC has been successfully applied in the isomeric separation of different *N*-glycopeptides [13,14]. Another chromatographic option for glycopeptide separation is hydrophilic interaction liquid chromatography (HILIC) [15]. HILIC is a powerful LC mode for the separation of polar analytes insufficiently retained in RP-LC [10,16]. Moreover, HILIC is also attractive in glycopeptides enrichment [17,18], and HILIC has been applied in the separation of the isomeric glycoforms differing only in linkage position and/or branching [15,19,20,21].

Changes in glycopeptide isomeric structures can also be used as potential biomarkers [9,22]. As such, Huang et al. have used HILIC to separate sialylated *N*-glycan isomers of fetuin differing only in α2–3 and α2–6 linkages [19]. Kozlik et al. separated core- and outer-arm-linked fucose into bi-antennary and tri-antennary glycoforms and the antennary position of sialic acid linked via α2–6 linkage of the hemopexin glycopeptides by HILIC [16].

In recent years, various commercial HILIC phases have been described with surface chemistries far more diverse than RP and which can provide substantially different selectivities. Among the most commonly and successfully used HILIC columns in glycopeptide separations, amide [23,24], ZIC-HILIC [25,26], and penta-HILIC [10,27] stand out for their resolution and versatility. For example, Gilar et al. successfully applied an amide column to the glyco-profiling of a therapeutic monoclonal antibody and proteins with several N-linked and O-linked glycosylation sites [28]. Although ZIC-HILIC columns are used for glycopeptide enrichment rather than for their separation [29,30,31], Hernandez-Hernandez et al. has used a ZIC-HILIC column to separate sialylated trisaccharide isomers of bovine caseinomacropeptide according to the sialic acid attachment site [32]. The Penta-HILIC column showed good separation potential for the analysis of hemopexin glycopeptides [16] and for the analysis of bovine fetuin glycopeptides and human IgG glycopeptides [19].

In this study, we investigated and compared the separation potential of three commercially available HILIC columns, i.e., HALO^®^ penta-HILIC, Glycan BEH Amide, and a zwitterionic type ZIC-HILIC column. The penta-HILIC uses proprietary bonding chemistry that includes five hydroxyl groups on the bonded ligand. The ZIC-HILIC column contains a zwitterionic sulfoalkylbetaine stationary phase covalently attached to porous silica. The amide column is based on the ethylene bridged hybrid (BEH) particle technology with trifunctionally-bonded amide phase. For comparison purposes, we studied glycopeptides prepared by trypsin digestion of well-characterized glycoproteins, i.e., human hemopexin and Immunoglobulin G. The findings of this study may therefore help to optimize the HILIC method for glycopeptide analysis.

## 2. Results and Discussion

In this study, we compared the separation potential of three different HILIC stationary phases in the analysis of complex *N*-glycopeptides of hemopexin and IgG glycoproteins. For this purpose, we selected the most intense tryptic glycopeptides of the aforementioned glycoproteins. All studied glycopeptides are outlined in Table 1. In this article, we use the following abbreviations for *N*-glycans: A2G2 for a bi-antennary glycan with two terminal galactoses, A3G3 for a tri-antennary glycan terminated with three galactoses, S for sialic acid, and F for fucose. For the chromatograms shown in the figures, we exported extracted-ion chromatograms (EIC) to OriginPro 8.5.0 (OriginLab Corporation, Northampton, MA, USA), normalized them to specific glycoform, and overlaid them. First, we optimized the gradient program to enhance, as much as possible, the resolution of the studied glycopeptides. For HALO^®^ penta-HILIC and ACQUITY UPLC Glycan BEH Amide, we used the same gradient program (see Instrumentation and experimental conditions). However, in the ZIC-HILIC column, a slower gradient program was applied to obtain satisfactory peak shapes and adequate retention and resolution.

### 2.1. Separation of Glycopeptides by HILIC

**Figure 1:**
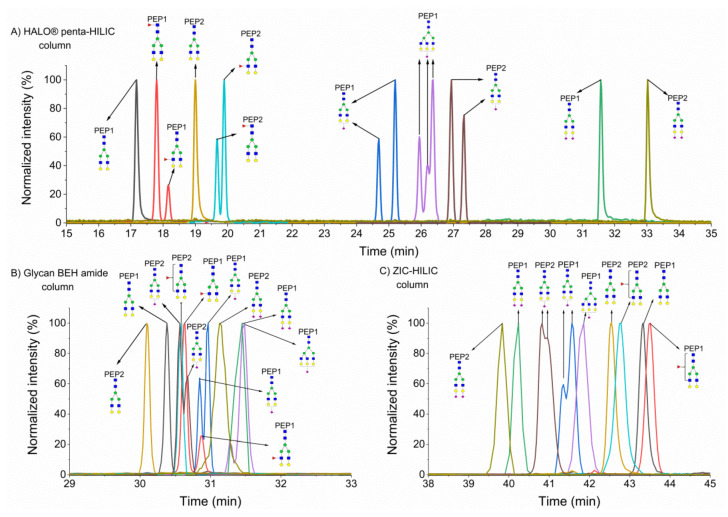
Separation of glycopeptides of hemopexin in HALO^®^ penta- hydrophilic interaction liquid chromatography (HILIC) (**A**), Glycan ethylene bridged hybrid (BEH) amide (**B**), and ZIC-HILIC (**C**) columns. PEP1 refers to SWPAVGN^187^CSSALR, and PEP2 to ALPQPQN^453^VTSLLGCTH.

As previously described, in HILIC, glycopeptide retention is mainly driven by the glycan moiety of the glycopeptide and by the polarity of the peptide backbone [26,33], increasing with the number of monosaccharide units in a glycan moiety [15,34], as shown in Figure 1. The A2G2 glycoform of the SWPAVGN^187^CSSALR peptide of hemopexin eluted as the first peak on a HALO^®^ penta-HILIC column (see Figure 1A). Adding a monosaccharide unit rendered the glycopeptide more polar/hydrophilic, thereby increasing the retention times. Sialic acid in a glycan moiety significantly prolonged the retention times of hemopexin glycopeptides, as shown in our previous study on HILIC in the nanoscale [15]. The separation of hemopexin glycopeptides in the Glycan BEH amide column is shown in Figure 1B. All glycopeptides eluted in a narrower retention window (30–31.7 min) in this column than in the HALO^®^ penta-HILIC column (17–34 min). Moreover, the Glycan BEH amide column provided different selectivity because the A2G2 glycoform of the ALPQPQN^453^VTSLLGCTH peptide eluted as the first peak in this column and as the second peak in the HALO^®^ penta-HILIC column. Additionally, the retention times of the sialylated glycopeptides of hemopexin did not increase considerably. In the ZIC-HILIC column (see Figure 1C), we observed the lowest retention for sialylated glycopeptides. This behavior can be explained by the electrostatic repulsion between the negatively charged sulfobetaine functional group and the negatively charged sialic groups of the glycans. Therefore, the lowest retention was observed for di-sialylated glycopeptides and the highest retention for non-sialylated glycopeptides, whereas the A2G2F1 glycoform of the SWPAVGN^187^CSSALR peptide eluted as the last peak in that column. All studied hemopexin glycopeptides eluted in the range of 39–44 min. Although the ZIC-HILIC column provided the longest retention for hemopexin glycopeptides, this column did not provide the best resolution among the columns tested in this study. The ZIC-HILIC column exhibited poor resolution between fucosylated and non-fucosylated glycoforms, as well as between A2G2S2 and A3G3S1 of the SWPAVGN^187^CSSALR peptide. The best chromatographic resolution (at almost baseline resolution) of all studied hemopexin glycopeptides was observed in the HALO^®^ penta-HILIC column. Several glycopeptides were co-eluted in the Glycan BEH amide column, namely: A2G2S1 of the ALPQPQN^453^VTSLLGCTH peptide and A2G2F1 of both peptides; A2G2S2 and A3G3S1 of the SWPAVGN^187^CSSALR peptide. In addition, Table S1 (Supplementary Materials) summarizes the intact masses and retention times of these glycopeptides in the studied HILIC columns.

**Figure 2:**
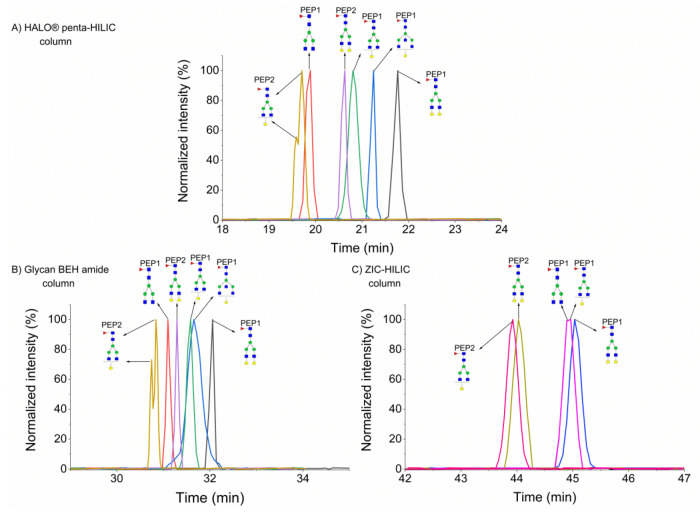
Separation of glycopeptides of IgG in HALO^®^ penta-HILIC (**A**), Glycan BEH amide (**B**), and ZIC-HILIC (**C**) columns. PEP1 refers to EEQYN^180^STYR and PEP2 to EEQFN^176^STFR.

As mentioned above, we chose two occupied peptides of the IgG glycoprotein, which only differ by two amino acids in the peptide sequence (IgG1: EEQYN^180^STYR and IgG2: EEQFN^176^STFR). Figure 2 shows that the A2G1F1 glycoform of the IgG2 peptide had the lowest retention in each HILIC column because tyrosine (Y) is more hydrophilic than phenylalanine (F); thus, the glycopeptides with this backbone elute earlier [19]. As shown in Figure 2A,B, this glycoform eluted in two peaks in the HALO^®^ penta-HILIC and Glycan BEH amide column, most likely due to a partial separation of the isobaric glycoforms resulting from the attachment of the galactose to each of the two antennae (α3 and α6), as previously described [19]. In the ZIC-HILIC column, this isomeric glycoforms eluted as one peak (see Figure 2C). Adding one galactose to the A2F1 or the A2G1F1 glycoform increased the retention time by approximately 1 min in the HALO^®^ penta-HILIC column. However, the addition of one *N*-acetylhexosamine to the center galactose molecule of the A2G1F1 glycopeptide increased the retention time by only 0.4 min because galactose (log P = −2.6) is more polar than *N*-acetylhexosamine (log P = −1.7) (https://www.ncbi.nlm.nih.gov/pccompound). A similar trend was observed in the Glycan BEH amide column, albeit with smaller shifts in retention times (see Figure 2B). Similarly to the separation of hemopexin glycopeptides, the ZIC-HILIC column provided the highest retention times and the poorest resolution of the studied IgG glycopeptides, as shown in Figure 2C. We observed a total co-elution of the A2F1 and A2G1F1 glycoforms of the IgG1 peptide. Moreover, we did not detect the G1A3F1 glycoform (for no plausible reason).

#### 2.1.1. Separation of Fucosylated Glycopeptides

**Figure 3:**
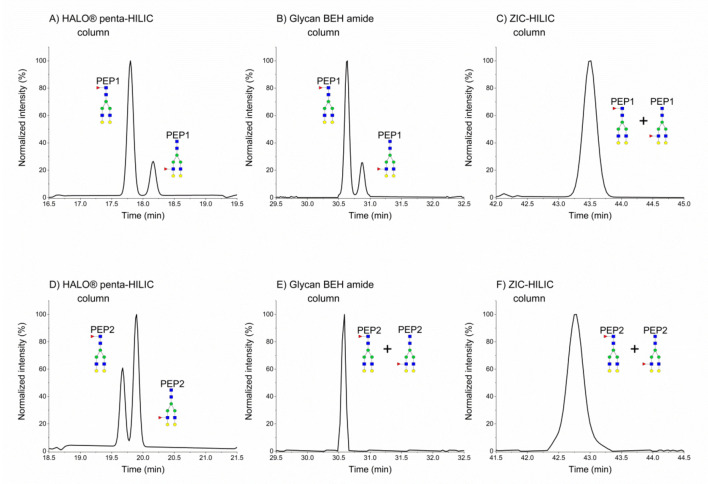
Normalized EIC chromatograms of A2G2F1 glycoforms of SWPAVGN^187^CSSALR (**A**–**C**) and ALPQPQN^453^VTSLLGCTH (**D**–**F**) in different HILIC columns. PEP1 refers to SWPAVGN^187^CSSALR, and PEP2 to ALPQPQN^453^VTSLLGCTH.

Enhanced fucosylation has been proposed as a biomarker for monitoring liver disease [35,36,37]. Accordingly, LC separation of fucosylated glycoform isomers can improve analytical workflow in disease progression. As we have shown previously, the HALO^®^ penta-HILIC column in nanoscale was able to separate mono-fucosylated glycopeptides of hemopexin, which only differed in the core- or outer arm-linked fucose positions [16]. We separated fucosylated glycopeptide isomers of hemopexin glycoproteins during the comparative study of different HILIC stationary phases. Figure 3A,D shows two well-separated peaks for hemopexin SWPAVGN^187^CSSALR and ALPQPQN^453^VTSLLGCTH in the HALO^®^ penta-HILIC column at chromatographic resolutions of 1.99 and 1.36, respectively. The assignment of core or outer arm fucosylation to chromatographic peaks was confirmed by exoglycosidase digestion in our previous study [16]. Our assumption was correct based on the fragmentation spectra of A2G2F1 with *m*/*z* 1058.45 (SWPAVGN^187^CSSALR) and 1168.84 (ALPQPQN^453^VTSLLGCTH). In core fucosylation, a fragment of m/z 1753.80 or m/z 2085.10 was identified (Figure S1 (Supplementary Materials)). This fragment was not detected in the fragmentation spectra of the outer arm-fucosylated glycopeptide. Although the separation in the SWPAVGN^187^CSSALR sequence of the A2G2F1 glycoform was better in the Glycan BEH Amide column (R = 1.72), we were unable to separate the isobaric glycoforms in the other hemopexin sequence (Figure 3E). Despite optimizing the gradient program for the ZIC-HILIC column, the core- and outer arm-fucosylated glycoforms were not separated, either in the SWPAVGN^187^CSSALR or in the ALPQPQN^453^VTSLLGCTH sequences (Figure 3C,F). Isomeric glycoforms eluted in a single broad peak. Moreover, only the HALO^®^ penta-HILIC column separated the fucosylated bi-antennary isomeric glycoforms of different peptide backbones.

#### 2.1.2. Separation of Sialylated Glycopeptides

**Figure 4:**
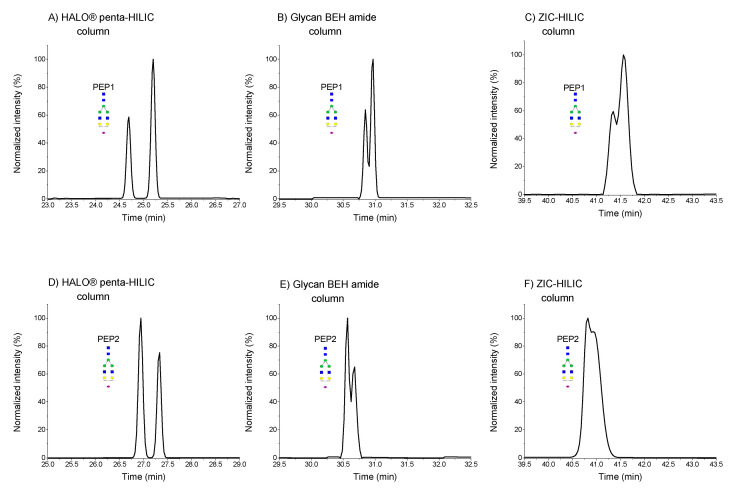
Normalized EIC chromatograms of A2G2S1 glycoforms of SWPAVGN^187^CSSALR (**A**–**C**) and ALPQPQN^453^VTSLLGCTH (**D**–**F**) in different HILIC columns. PEP1 refers to SWPAVGN^187^CSSALR, and PEP2 to ALPQPQN^453^VTSLLGCTH.

As shown in Figure 4, the EIC chromatograms of mono-sialylated glycoforms of hemopexin have multiple isobaric peaks. Based on our previous study [16], mono-sialylated hemopexin glycoforms contain isomers that differ in the localization of sialic acid on different arms (α3 antenna or α6 antenna). In each column, we obtained different resolutions, achieving the best resolution in the HALO^®^ penta-HILIC column, wherein the two isobaric glycoforms were separated with a resolution of 3.09 for the SWPAVGN^187^CSSALR and of 2.24 for the ALPQPQN^453^VTSLLGCTH sequence. Neither the Glycan BEH amide column nor the ZIC-HILIC column was able to baseline separate these glycoforms of both peptides (Figure 4B–F). The Glycan BEH amide column provided a resolution of 1.02 for the SWPAVGN^187^CSSALR and of 0.82 for the ALPQPQN^453^VTSLLGCTH peptide. The ZIC-HILIC column provided the worst resolutions. The resolution was 0.74 for the SWPAVGN^187^CSSALR peptide and 0.43 for the ALPQPQN^453^VTSLLGCTH peptide.

The separation of the A3G3S1 glycoform of the SWPAVGN^187^CSSALR peptide in the HALO^®^ penta-HILIC column is shown in Figure S2A (Supplementary Materials), displaying three peaks with resolution R_1,2_ = 1.28 and R_2,3_ = 0.80. In the Glycan BEH amide column, only two peaks were separated (Figure S2B (Supplementary Materials)), in contrast to only one peak in the ZIC-HILIC column (Figure S2C (Supplementary Materials)), most likely as a result of partial isomer separation of the α2–6 sialic acid (either the unbranched α6-antenna or the branched α3-antenna with β2- or β4-branching). At this point, we are still unable to elucidate which peak corresponds to which isomer.

### 2.2. Column Temperature Effect on the Separation of Glycopeptides Isomers

In line with Mechref et al., who studied the column temperature effect on the isomeric separation of *N*-glycopeptides by PGC [13], we tested three different column temperatures, i.e., 40, 50, and 60 °C. The retention times and resolutions of all studied isomeric glycopeptides separated in the three HILIC columns are shown in Table 2. In most cases, the retention time decreased with an increase in temperature.

HALO^®^ penta-HILIC column: For hemopexin with the glycan composition of A2G2F1, the resolution decreased with an increase in temperature for the SWPAVGN^187^CSSALR and ALPQPQN^453^VTSLLGCTH sequences (Table 2). In turn, for the mono-sialylated glycoforms of hemopexin, the resolution increased with temperature, albeit only for the peptide sequence ALPQPQN^453^VTSLLGCTH. For the other hemopexin glycopeptide, the resolution decreased at 50 °C column temperature but increased at 60 °C. The column temperature also affected the separation of the A3G3S1 glycoform of hemopexin (sequence SWPAVGN^187^CSSALR). At 40 °C, we were able to partly separate the three isobaric glycoforms of A3G3S1. However, at 50 °C, the second peak started co-eluting with the third peak, and the second and the third peaks fully co-eluted at 60 °C, as shown in Figure S3 (Supplementary Materials). In the separation of the A2G1F glycoform of IgG2 (EEQFN^176^STFR), the resolution slightly improved when increasing the column temperature to 50 °C, but worsened at 60 °C.

Glycan BEH Amide column: The resolution of fucosylated bi-antennary hemopexin with the sequence SWPAVGN^187^CSSALR improved by increasing the column temperature to 50 °C, but worsened at 60 °C. We observed the same tendency for the A2G2S1 glycoform with the sequence ALPQPQN^453^VTSLLGCTH. Conversely, the resolution slightly increased at 50 °C and 60 °C for mono-sialylated bi-antennary hemopexin with the SWPAVGN^187^CSSALR sequence. In the separation of mono-sialylated tri-antennary glycan (A3G3S1) of hemopexin with the SWPAVGN^187^CSSALR sequence, the resolution worsened with the increase in column temperature. The resolution of the A2G1F isomer of IgG2 with the EEQFN^176^STFR sequence improved with the increase in temperature.

SeQuant ZIC-HILIC column: In the isomeric separation of the A2G2S1 glycoform of the SWPAVGN^187^CSSALR peptide, increasing the temperature improved the chromatographic resolution (see Figure S4 (Supplementary Materials)). We also observed a slightly better resolution for the A2G2S1 glycoform of the ALPQPQN^453^VTSLLGCTH peptide, as shown in Figure S4 (Supplementary Materials). However, the increased temperature did not affect the separation of the outer arm, the core fucosylation of the studied hemopexin glycopeptides, or the A2G1F glycoform of IgG2.

The increased temperature helped in the isomeric separation of some glycoforms but not others. The separation mechanism of HILIC is very complex, and we cannot reach a general conclusion of the column temperature effect on isomeric separation in HILIC at this point. More detailed studies are necessary to improve our understanding of the impact of column temperature on the LC separation of isomeric glycopeptides by HILIC.

## 3. Materials and Methods

### 3.1. Chemicals

Acetonitrile (LC-MS grade), formic acid (LC-MS grade), iodoacetamid (purity ≥ 99%), dithiotreitol (purity ≥ 99%), and SOLu-Trypsin were purchased from Sigma-Aldrich (St.Louis, MO, USA), as well as the IgG standard from human serum (UniProtKB ID, IgG1, P01857; IgG2, P01859). The hemopexin standard from human plasma (P02790) was purchased from Athens Research and Technology, Inc. (Athens, GA, USA). Water (LC-MS grade), ammonium hydrogen carbonate (LC-MS grade), and acetic acid (LC-MS grade) were supplied by Merck (Darmstadt, Germany). α2–3,6,8,9 neuraminidase (P0722S), and GlycoBuffer 1 was purchased from New England BioLabs (Ipswich, MA, USA).

### 3.2. Sample Preparation

The glycopeptide standards were prepared by trypsin digestion of 30 µL of hemopexin (10 µg µL^−1^) and 100 µL of IgG (2 µg µL^−1^) according to a previously described protocol [38]. After trypsin digestion, the samples were desalted using solid-phase extraction (SPE) on a Sep-Pak Vac C18 cartridge (Waters, Milford). The following desalting procedure was used: Condition step: 2 × 1 mL of 100% acetonitrile and 1 mL of 2% acetic acid; loading step; washing step: 0.5 mL of 2% acetic acid; elution step: 0.5 mL of 70% acetonitrile with 2% acetic acid. 100 µL of hemopexin digest was mixed with 100 µL of IgG digest. In total, 100 µL of the mixture was subjected to desialylation by adding 14 μL of GlycoBuffer 1 and 14 μL of neuraminidase overnight at 37 °C. The desialylated sample was desalted, as mentioned above, and eluted in 0.3 mL 70% acetonitrile with 2% acetic acid. Then, 75 µL of the glycoprotein tryptic digest mixture was mixed with 75 µL of the desialylated sample. The mixture was evaporated and reconstituted in 65% acetonitrile with 0.1% formic acid, analyzing 1 µL by HILIC.

### 3.3. Instrumentation and Experimental Conditions

The measurements were performed on an Agilent 1290 Infinity II LC System with a binary pump (Agilent Technologies, Inc., Waldbronn, Germany) coupled with maXis™ Q-TOF mass spectrometer (Bruker Daltonics, Bremen, Germany). The glycopeptide separation was tested in three different HILIC columns: HALO^®^ penta-HILIC (2.1 × 150 mm; 2.7 µm; Advanced Materials Technology, Wilmington, Delaware, USA), ACQUITY UPLC Glycan BEH Amide Column (2.1 × 150 mm; 1.7 µm; Waters Corporation, Milford, MA, USA) and SeQuant ZIC-HILIC (2.1 × 150 mm; 3.5 µm; Merck, Darmstadt, Germany). The mobile phase consisted of 0.1% formic acid in water (A) and 0.1% formic acid in acetonitrile (B). The gradient program was optimized to enhance, as much as possible, the resolution of all glycopeptides analyzed in this study. In the case of HALO^®^ penta-HILIC and ACQUITY UPLC Glycan BEH Amide, the following gradient ((min)/% B) was used: 0/80, 10/80, 25/65, 35/40, 40/40, 43/80, 55/80. For the SeQuant ZIC-HILIC column, a shallower gradient program was used: 0/80, 10/80, 35/65, 45/40, 55/40, 58/40, 70/80. The flow rate was 0.3 mL min^−1^ for every column, and the column temperature was maintained at 40 °C (if not stated otherwise).

### 3.4. Mass Spectrometry Settings

The glycopeptides were analyzed in a data-dependent acquisition mode. The Hystar 3.2 and otofControl 4.0 software (Bruker Daltonics, Bremen, Germany) were used for data acquisition. The acquired MS data were analyzed using Bruker DataAnalysis 4.4. The experimental conditions for the source were set as follows: End plate offset 500 V, capillary voltage 3500 V, dry gas 8 L min^−1^ with 250 °C. For mass spectrometer calibration, an ESI-L-low concentration tune mix (Agilent Technologies, Santa Clara, California) was used in a mid-positive calibration mode. The MS spectra were acquired at a mass range of m/z 100–3000 at a spectra rate of 2 Hz with precursor selection in the range of 600–3000 m/z, setting the preferred charges states between +2 and +4. After MS acquisition, stepping-energy the five most intense precursors were subjected to collision-induced dissociation (CID), setting the collision energy to 70 eV and collecting mixed energy spectra at 100% collision energy for 50% of the cycle time and 50% collision energy for other 50% of the cycle time, with the product-ion spectra rate ranging from 4 to 16 Hz, depending on precursor intensity. In this operation mode, ion counts from each subdivision were summed together. Glycopeptides were identified manually from the precursor masses and CID spectra. The fragmentation spectra of all the studied glycopeptides are shown in Figure S1 (Supplementary Materials).

## 4. Conclusions

In this study, we compared three different HILIC stationary phases, i.e., HALO^®^ penta-HILIC, Glycan BEH Amide, and ZIC-HILIC in the separation of complex *N*-glycopeptides of hemopexin and IgG glycoproteins. We showed the effect of the composition of the glycans on the retention and separation of glycopeptides. Adding a monosaccharide unit to a glycan moiety enhances glycopeptide retention. Sialic acid in a glycan moiety significantly prolonged the retention times of hemopexin glycopeptides in the HALO^®^ penta-HILIC column. In contrast, in the Glycan BEH Amide column, this effect was unremarkable. In the ZIC-HILIC column, we observed electrostatic repulsion between the negatively charged sulfobetaine functional group and the negatively charged sialic groups of the glycans, thereby decreasing retention. The HALO^®^ penta-HILIC column provided the best separation of the studied glycopeptides and the ZIC-HILIC column the worst. We also demonstrated the potential of the HILIC columns tested in this study for the isomeric separation of fucosylated and sialylated glycoforms. The HALO^®^ penta-HILIC column provided good separation of core- and outer arm-linked fucose of the A2G2F1 glycoform of both hemopexin peptides. The Glycan BEH Amide column was able to separate only the A2G2F1 isomeric glycoforms of the SWPAVGN^187^CSSALR peptide, whereas the ZIC-HILIC column did not separate any A2G2F1 isomers. The HALO^®^ penta-HILIC column showed the best separation of sialylated glycoforms, followed by the Glycan BEH Amide column, whereas the ZIC-HILIC column provided a very poor chromatographic resolution. Choosing the right HILIC stationary phase is a crucial step when optimizing the HILIC method. Therefore, the findings of this study may help to develop the HILIC method for glycopeptide analysis because the HILIC technique is a potentially useful tool for the comprehensive characterization of glycoproteins and their isomers.

## Figures and Tables

**Table 1:** The studied glycopeptides of hemopexin and IgG. Symbols: 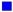, *N*-acetylglucosamine (GlcNAc); 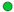, Mannose (Man); 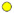, Galactose (Gal); 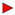, Fucose (Fuc); 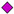, Sialic acid.

Hemopexin					
SWPAVGN^187^CSSALR	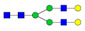 A2G2	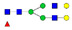 A2G2F1	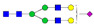 A2G2S1	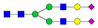 A2G2S2	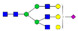 A3G3S1
ALPQPQN^453^VTSLLGCTH	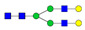 A2G2	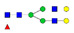 A2G2F1	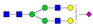 A2G2S1	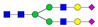 A2G2S2	
IgG					
EEQYN^180^STYR (IgG1)	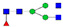 A2F1	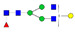 A2G1F	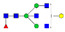 G1A3F1	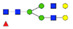 A2G2F1	
EEQFN^176^STFR (IgG2)	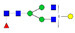 A2G1F	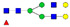 A2G2F1			

A2G2 = bi-antennary glycan with two terminal galactoses; A3G3 = tri-antennary glycan terminated with three galactoses; S = sialic acid; F = fucose.

**Table 2:** Chromatographic parameters: *t*_R_, retention time; *R*, resolution of the HILIC separation of glycopeptide isomers of hemopexin and IgG. ND means not detected analyte.

	HALO^®^ Penta-HILIC						Glycan BEH Amide						ZIC-HILIC					
	Column Temperature																	
	40 °C		50 °C		60 °C		40 °C		50 °C		60 °C		40 °C		50 °C		60 °C	
**Hemopexin** SWPAVGN^187^CSSALR	*t*_R_	*R*	*t*_R_	*R*	*t*_R_	*R*	*t*_R_	*R*	*t*_R_	*R*	*t*_R_	*R*	*t*_R_	*R*	*t*_R_	*R*	*t*_R_	*R* *r*
A2G2F1 (core)	17.8	1.99	17.5	1.85	17.1	1.81	30.6	1.71	30.5	2.34	30.3	1.82	43.5	−	43.1	−	43.0	−
A2G2F1 (outer arm)	18.2		17.8		17.5		30.9		30.8		30.6							
A2G2S1 (isomer 1)	24.7	3.09	24.4	2.89	24.1	3.33	30.8	1.02	30.7	1.05	30.6	1.12	41.3	0.74	41.0	0.86	41.1	0.89
A2G2S1 (isomer 2)	25.2		24.9		24.7		30.9		30.9		30.7		41.6		41.3		41.4	
A3G3S1 (isomer 1)	25.9	1.28 1.80	25.7	0.39 0.37	25.4	1.33	31.3	1.23	31.3	0.74	31.1	0.73	41.8	−	41.7	ND		
A3G3S1 (isomer 2)	26.2		26.0		25.8		31.5		31.4		31.2							
A3G3S1 (isomer 3)	26.4		26.1		ND		ND		ND		ND		ND		ND		ND	
ALPQPQN^453^VTSLLGCTH																		
A2G2F1 (core)	19.7	1.36	19.3	1.09	18.9	1.07	30.6	−	30.5	−	30.2	−	42.7	−	ND		ND	
A2G2F1 (outer arm)	19.9		19.6		19.2													
A2G2S1 (isomer 1)	26.9	2.24	26.7	2.72	26.3	3.11	30.5	0.82	30.4	1.01	30.3	0.99	40.8	0.43	40.6	0.50	40.5	0.61
A2G2S1 (isomer 2)	27.3		27.1		26.8		30.6		30.6		30.4		40.9		40.7		40.7	
**IgG2** EEQFN^176^STFR																		
A2G1F (isomer 1)	19.5	0.74	19.4	0.80	19.2	0.73	30.8	0.90	30.7	0.97	30.5	1.02	43.9	−	43.7	−	43.5	−
A2G1F (isomer 2)	19.7		19.6		19.4		30.9		30.8		30.6							

## Supplementary Materials

The following are available online, Fragmentation spectra of the A2G2F1 glycoform of the SWPAVGN^187^CSSALR and ALPQPQN^453^VTSLLGCTH peptide in the HALO^®^ penta-HILIC column (Figure S1 (Supplementary Materials)). Separation of the A3G3S1 glycoform of the SWPAVGN^187^CSSALR peptide of hemopexin in different HILIC columns (Figure S2 (Supplementary Materials)) and column temperature effect on the separation of this glycoform in the HALO^®^ penta-HILIC column (Figure S3 (Supplementary Materials)). Separation of the A2G2S1 glycoform of hemopexin in both peptide sequences at different column temperatures in the ZIC-HILIC column (Figure S4 (Supplementary Materials)).
