# Supplementary material for: Comparison of Different HILIC Stationary Phases in the Separation of Hemopexin and Immunoglobulin G Glycopeptides and Their Isomers
Source: Molecules. 2020 Oct 13;25(20):4655. doi: 10.3390/molecules25204655 (PMC7594091; doi:10.3390/molecules25204655)
Supplement: Supplementary file 1 [file molecules-25-04655-s001.pdf]

Supporting Information

Comparison of different HILIC stationary phases in the separation of hemopexin and immunoglobulin G glycopeptides and their isomers

Katarina Molnarova [1], Petr Kozlik [1,*]

[1] Department of Analytical Chemistry, Faculty of Science, Charles University, Hlavova 8, 128 43 Prague 2, Czech Republic; katarina.molnarova@natur.cuni.cz (M.K.) ; kozlik@natur.cuni.cz (P.K.)

* Correspondence

RNDr. Petr Kozlík, Ph.D.

Charles University

Hlavova 8

Prague 2 -128 43

Czech Republic

Email: kozlik@natur.cuni.cz

Tel: 221 951 216

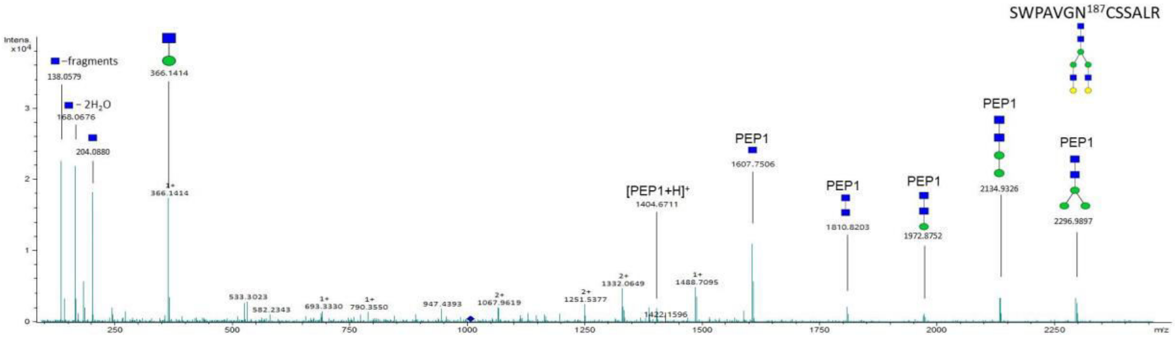
SWPAVGN187CSSALR
Intens. x10^4
–fragments
138.0579
– 2H2O
168.0676
204.0880
366.1414
1+
366.1414
533.3023
582.2343
1+
693.3330
1+
790.3550
947.4393
2+
1067.9619
2+
1251.5377
2+
1332.0649
[PEP1+H]+
1404.6711
1422.1596
1+
1488.7095
PEP1
1607.7506
PEP1
1810.8203
PEP1
1972.8752
PEP1
2134.9326
PEP1
2296.9897
m/z

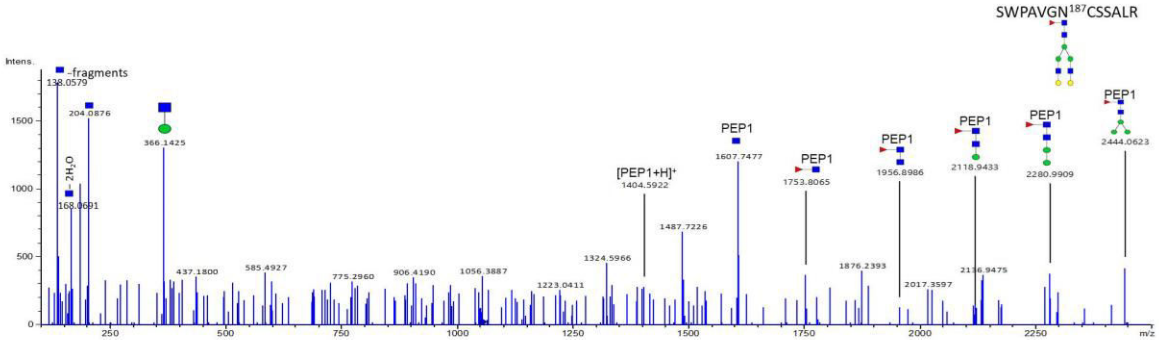
SWPAVGN187CSSALR
Intens.
–fragments
138.0579
– 2H2O
168.0691
204.0876
366.1425
437.1800
585.4927
775.2960
906.4190
1056.3887
1223.0411
1324.5966
[PEP1+H]+
1404.5922
1487.7226
PEP1
1607.7477
PEP1
1753.8065
1876.2393
PEP1
1956.8986
2017.3597
PEP1
2118.9433
2136.9475
PEP1
2280.9909
PEP1
2444.0623
m/z

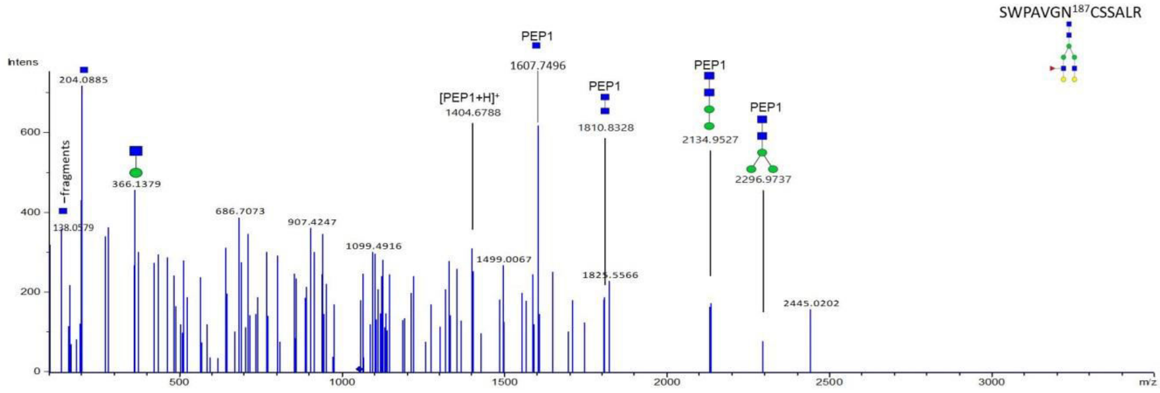
SWPAVGN187CSSALR
Intens
204.0885
–fragments
138.0579
366.1379
686.7073
907.4247
1099.4916
[PEP1+H]+
1404.6788
1499.0067
PEP1
1607.7496
PEP1
1810.8328
1825.5566
PEP1
2134.9527
PEP1
2296.9737
2445.0202
m/z

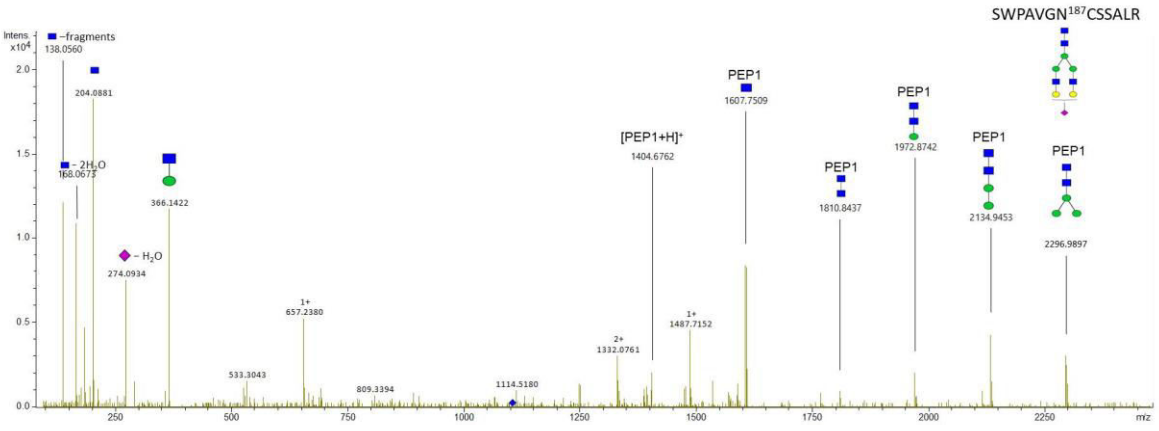
SWPAVGN187CSSALR
Intens. x10^4
–fragments
138.0560
204.0881
– 2H2O
168.0673
366.1422
– H2O
274.0934
533.3043
1+
657.2380
809.3394
1114.5180
2+
1332.0761
[PEP1+H]+
1404.6762
1+
1487.7152
PEP1
1607.7509
PEP1
1810.8437
PEP1
1972.8742
PEP1
2134.9453
PEP1
2296.9897
m/z

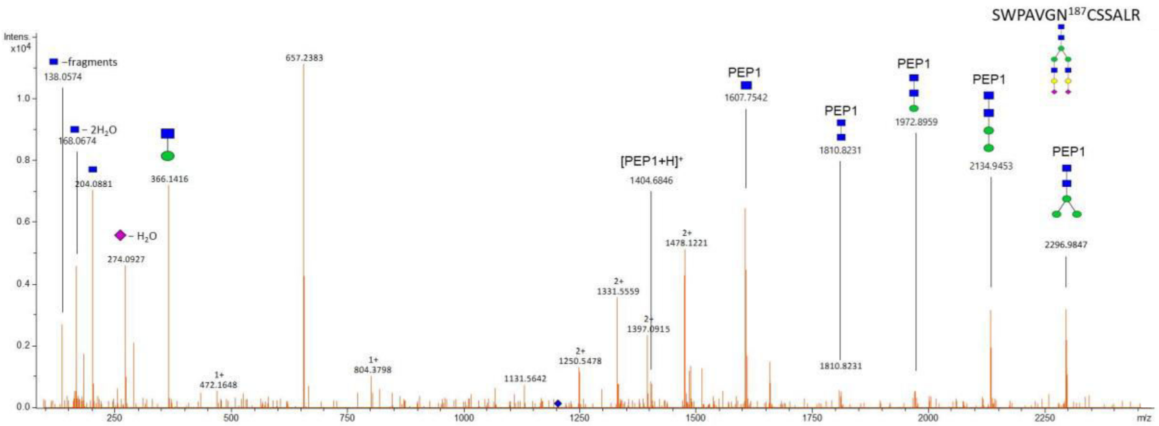
SWPAVGN187CSSALR
Intens. x10^4
–fragments
138.0574
– 2H2O
168.0674
204.0881
366.1416
– H2O
274.0927
657.2383
1+
472.1648
1+
804.3798
1131.5642
2+
1250.5478
2+
1331.5559
2+
1397.0915
[PEP1+H]+
1404.6846
2+
1478.1221
PEP1
1607.7542
PEP1
1810.8231
1810.8231
PEP1
1972.8959
PEP1
2134.9453
PEP1
2296.9847
m/z

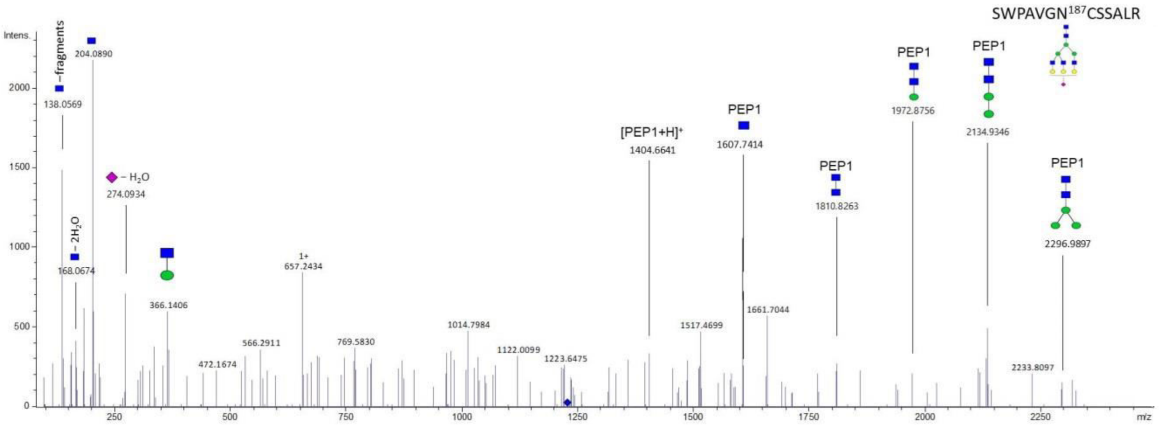
SWPAVGN187CSSALR
Intens.
–fragments
138.0569
– 2H2O
168.0674
204.0890
– H2O
274.0934
366.1406
472.1674
566.2911
1+
657.2434
769.5830
1014.7984
1122.0099
1223.6475
[PEP1+H]+
1404.6641
1517.4699
PEP1
1607.7414
1661.7044
PEP1
1810.8263
PEP1
1972.8756
PEP1
2134.9346
2233.8097
PEP1
2296.9897
m/z

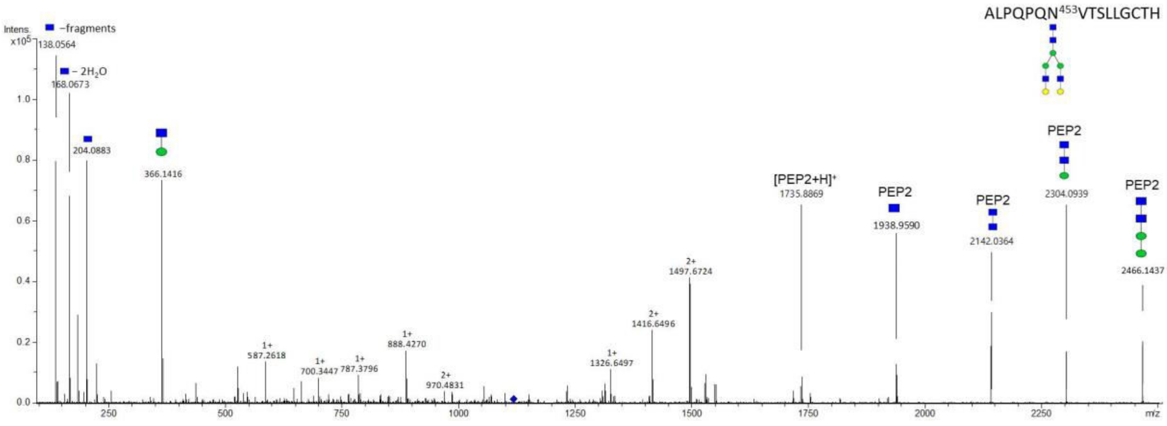
ALPQPQN453VTSLLGCTH
Intens. x10^5
–fragments
138.0564
– 2H2O
168.0673
204.0883
366.1416
1+
587.2618
1+
700.3447
1+
787.3796
1+
888.4270
2+
970.4831
1+
1326.6497
2+
1416.6496
2+
1497.6724
[PEP2+H]+
1735.8869
PEP2
1938.9590
PEP2
2142.0364
PEP2
2304.0939
PEP2
2466.1437
m/z

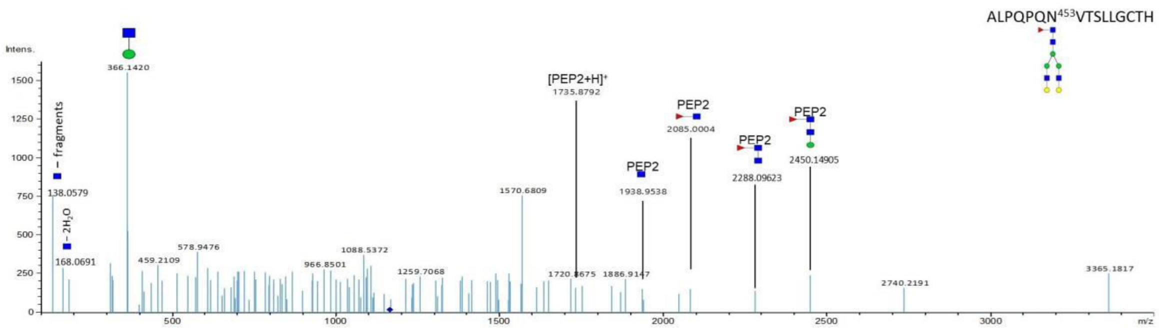
ALPQPQN453VTSLLGCTH
Intens.
366.1420
– fragments
138.0579
– 2H2O
168.0691
459.2109
578.9476
966.8501
1088.5372
1259.7068
1570.6809
[PEP2+H]+
1735.8792
1720.8675
1886.9147
PEP2
1938.9538
PEP2
2085.0004
PEP2
2288.09623
PEP2
2450.14905
2740.2191
3365.1817
m/z

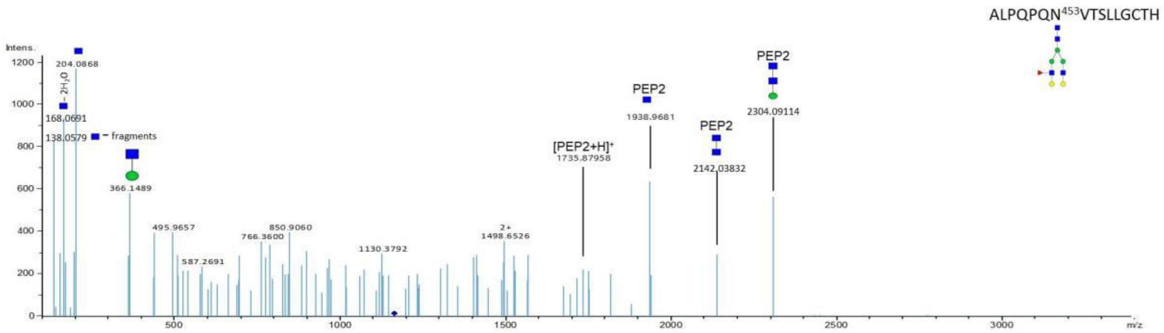

ALPQPQN453VTSLLGCTH
Intens.
- 2H2O
- fragments
204.0868
168.0691
138.0579
366.1489
495.9657
587.2691
766.3600
850.9060
1130.3792
2+
1498.6526
[PEP2+H]+
1735.87958
PEP2
1938.9681
PEP2
2142.03832
PEP2
2304.09114
m/z

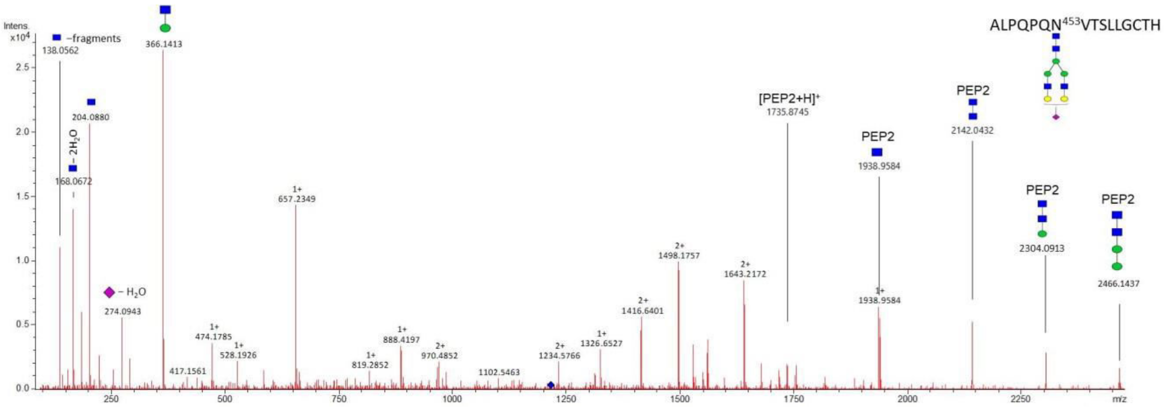

ALPQPQN453VTSLLGCTH
Intens.
x10^4
–fragments
138.0562
366.1413
204.0880
–2H2O
168.0672
– H2O
274.0943
1+
657.2349
1+
474.1785
417.1561
1+
528.1926
1+
819.2852
1+
888.4197
2+
970.4852
1102.5463
2+
1234.5766
1+
1326.6527
2+
1416.6401
2+
1498.1757
2+
1643.2172
[PEP2+H]+
1735.8745
PEP2
1938.9584
1+
1938.9584
PEP2
2142.0432
PEP2
2304.0913
PEP2
2466.1437
m/z

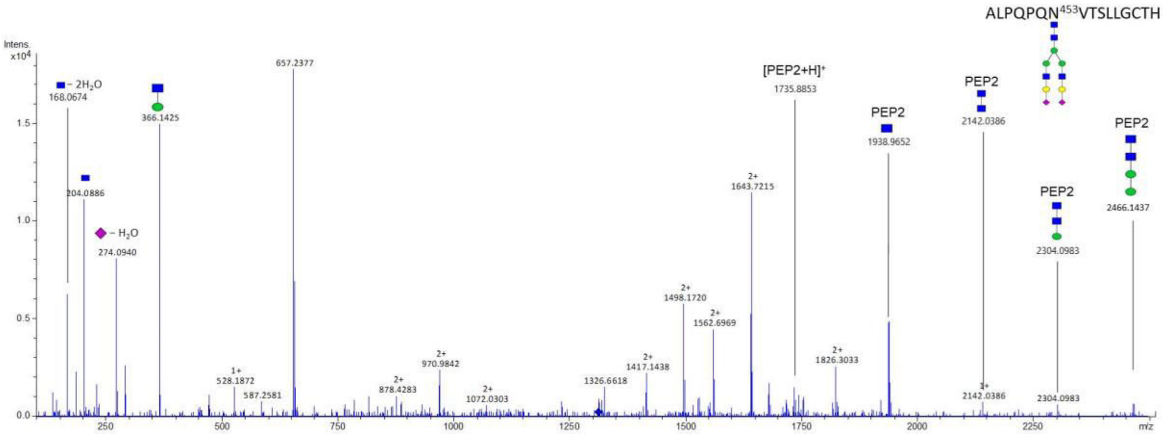

ALPQPQN453VTSLLGCTH
Intens.
x10^4
– 2H2O
168.0674
366.1425
657.2377
204.0886
– H2O
274.0940
1+
528.1872
587.2581
2+
878.4283
2+
970.9842
2+
1072.0303
1326.6618
2+
1417.1438
2+
1498.1720
2+
1562.6969
2+
1643.7215
[PEP2+H]+
1735.8853
2+
1826.3033
PEP2
1938.9652
PEP2
2142.0386
1+
2142.0386
PEP2
2304.0983
2304.0983
PEP2
2466.1437
m/z

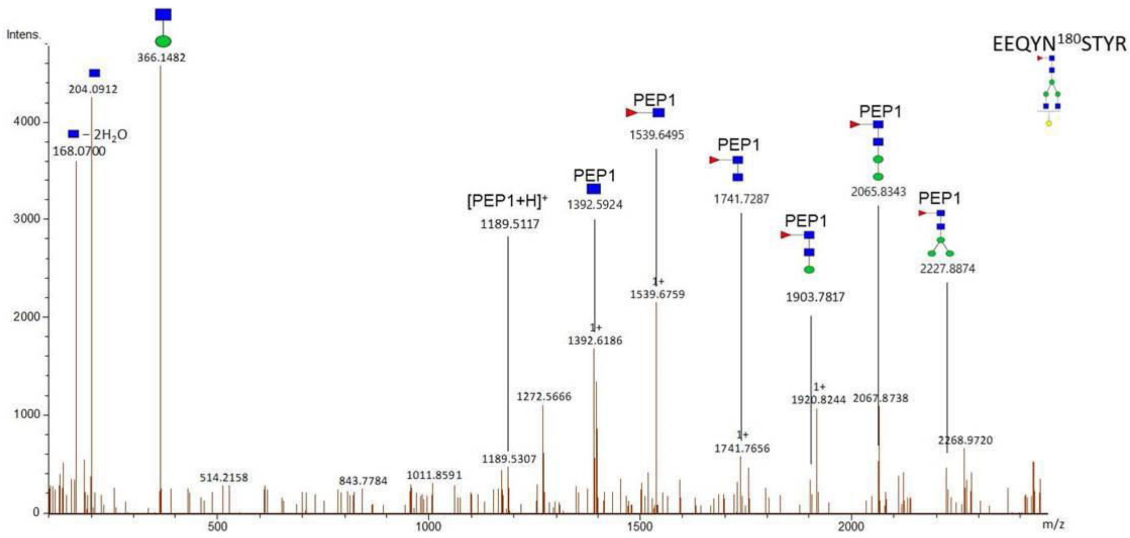

EEQYN180STYR
Intens.
366.1482
204.0912
– 2H2O
168.0700
514.2158
843.7784
1011.8591
[PEP1+H]+
1189.5117
1189.5307
1272.5666
PEP1
1392.5924
1+
1392.6186
PEP1
1539.6495
1+
1539.6759
PEP1
1741.7287
1+
1741.7656
PEP1
1903.7817
1+
1920.8244
PEP1
2065.8343
2067.8738
PEP1
2227.8874
2268.9720
m/z

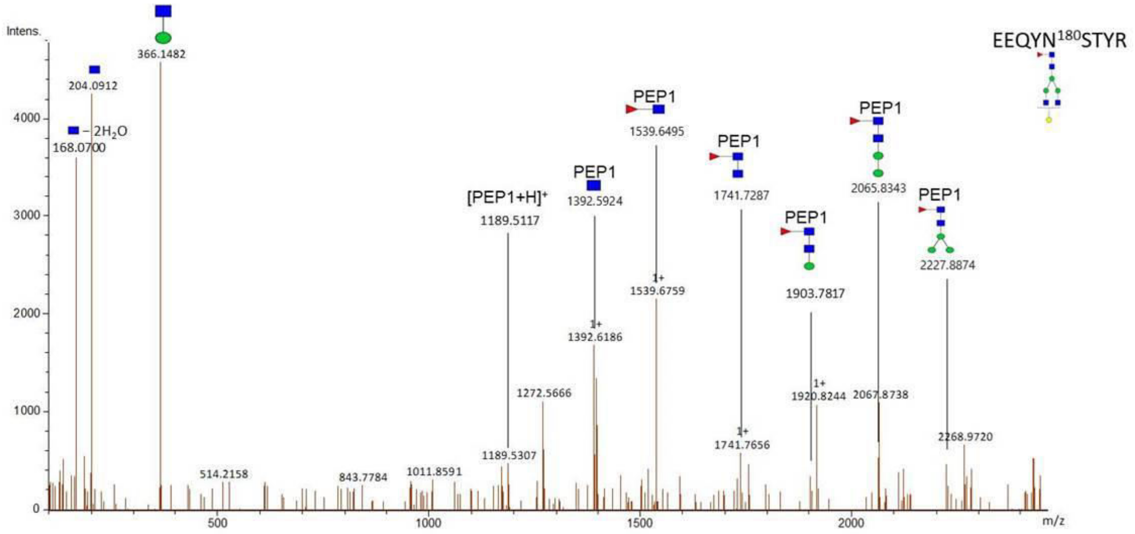
Intens.
EEQYN180STYR
366.1482
204.0912
− 2H2O
168.0700
PEP1
1539.6495
PEP1
1741.7287
PEP1
2065.8343
[PEP1+H]+
1189.5117
PEP1
1392.5924
PEP1
2227.8874
PEP1
1903.7817
1+
1539.6759
1+
1392.6186
1272.5666
1+
1920.8244
2067.8738
1+
1741.7656
2268.9720
514.2158
843.7784
1011.8591
1189.5307
m/z

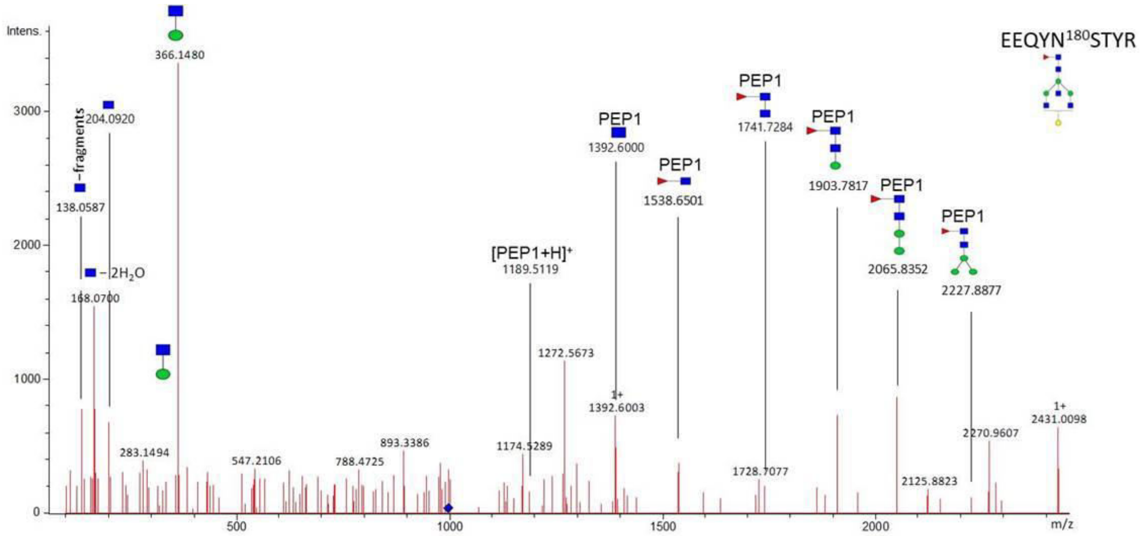
Intens.
EEQYN180STYR
366.1480
−fragments
204.0920
138.0587
− 2H2O
168.0700
PEP1
1392.6000
PEP1
1538.6501
PEP1
1741.7284
PEP1
1903.7817
PEP1
2065.8352
PEP1
2227.8877
[PEP1+H]+
1189.5119
1272.5673
1+
1392.6003
1+
2431.0098
2270.9607
283.1494
547.2106
788.4725
893.3386
1174.5289
1728.7077
2125.8823
m/z

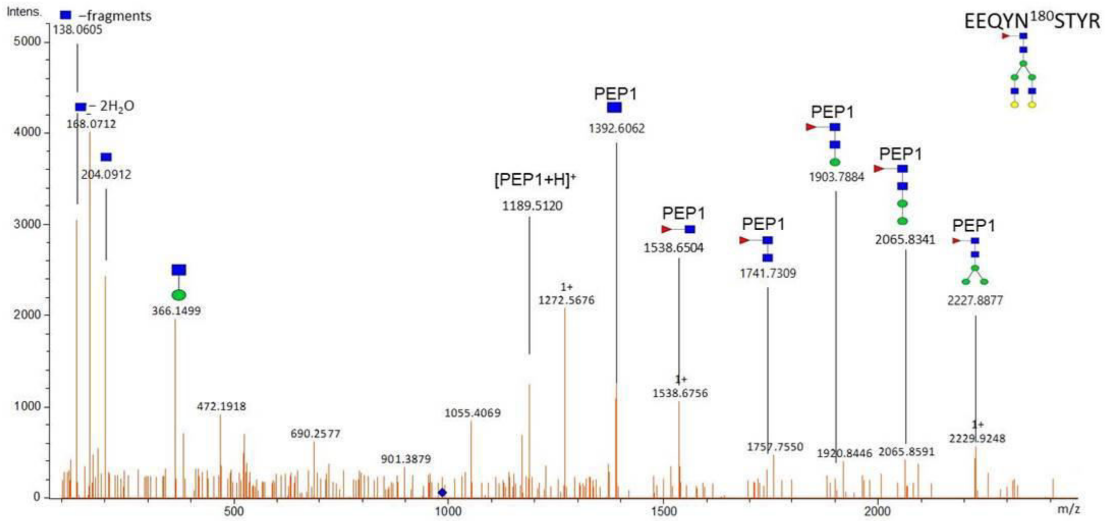
Intens.
−fragments
138.0605
EEQYN180STYR
− 2H2O
168.0712
204.0912
PEP1
1392.6062
[PEP1+H]+
1189.5120
PEP1
1538.6504
PEP1
1741.7309
PEP1
1903.7884
PEP1
2065.8341
PEP1
2227.8877
366.1499
1+
1272.5676
1+
1538.6756
472.1918
690.2577
901.3879
1055.4069
1757.7550
1920.8446
2065.8591
1+
2229.9248
m/z

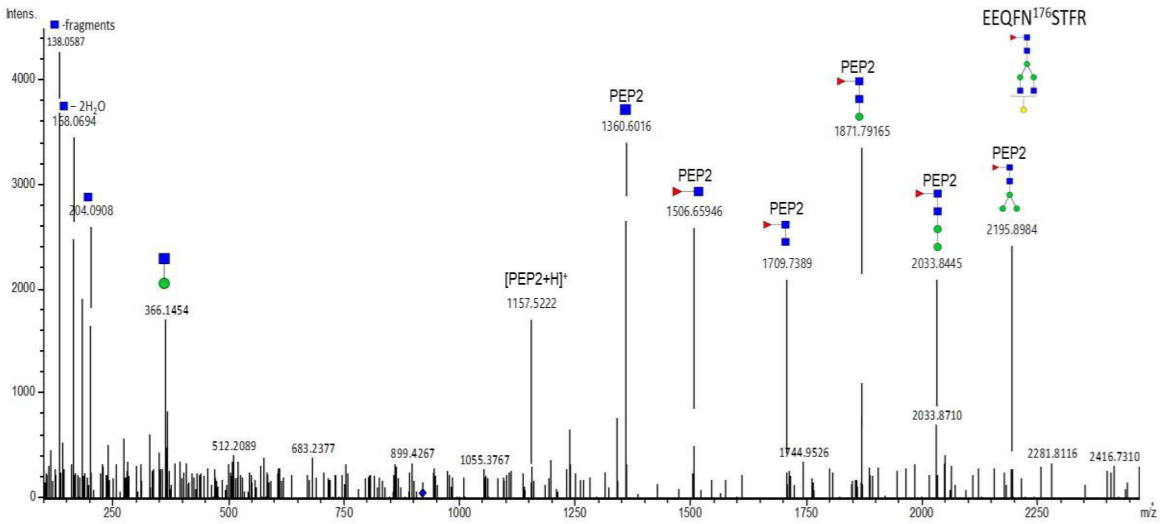

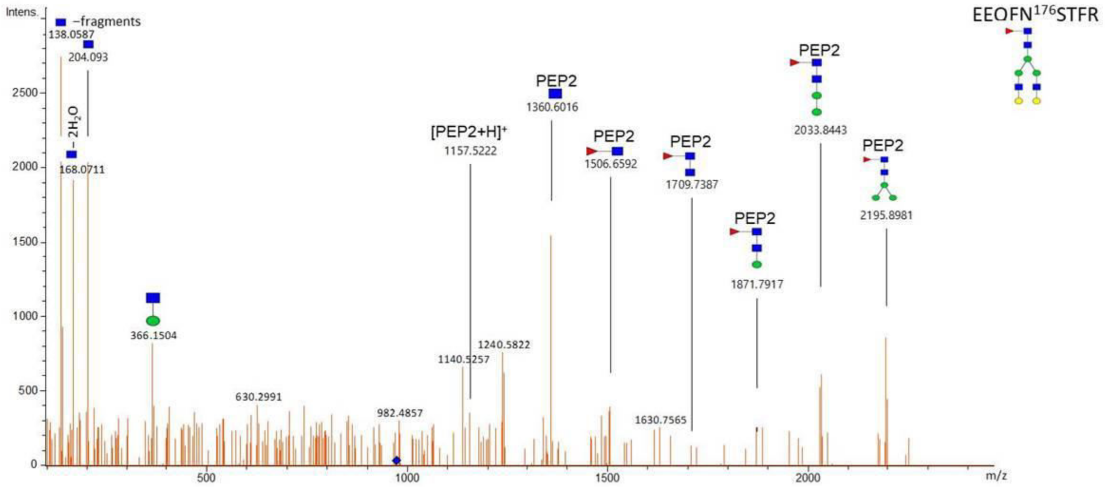

Figure S1 Fragmentation spectra of all studied glycopeptides in HALO® penta-HILIC column.

Table S1 Observed monoisotopic *m/z* for all studied analytes and their rentention times, t_R, in different HILIC columns. ND means not detected analyte.

Analyte	Column			
**Hemopexin** SWPAVGN187CSSALR	Observed *m/z* (Charge)	HALO® penta-HILIC t_R	Glycan BEH Amide t_R	ZIC-HILIC t_R
A2G2	1009.7550 (3+)	17.2	30.4	43.3
A2G2F1 (core)	1058.4409 (3+)	17.8	30.6	43.5
A2G2F1 (outer arm)		18.2	30.9	
A2G2S1 (isomer 1)	1106.7868 (3+)	24.7	30.8	41.3
A2G2S1 (isomer 2)		25.2	30.9	41.6
A2G2S2	1203.8186 (3+)	31.6	31.4	40.2
A3G3S1 (isomer 1)	1228.4975 (3+)	25.9	31.3	41.9
A3G3S1 (isomer 2)		26.2	31.5	ND
A3G3S1 (isomer 3)		26.4	ND	ND
ALPQPQN453VTSLLGCTH				
A2G2	1120.1586 (3+)	19.0	30.1	42.5
A2G2F1 (core)	1168.8445 (3+)	19.7	30.6	42.7
A2G2F1 (outer arm)		19.9		
A2G2S1 (isomer 1)	1217.1904 (3+)	26.9	30.5	40.8
A2G2S1 (isomer 2)		27.3	30.6	40.9
A2G2S2	1314.2222 (3+)	33.0	31.1	39.8
**IgG1** EEQYN180STYR				
A2F1	878.6868 (3+)	19.9	31.1	44.9
A2G1F	932.7044 (3+)	20.8	31.6	44.9
G1A3F1	1000.3975 (3+)	21.2	31.7	ND
A2G2F1	986.7220 (3+)	21.7	32.1	45
**IgG2** EEQFN176STFR				
A2G1F (isomer 1)	922.0411 (3+)	19.5	30.8	43.9
A2G1F (isomer 2)		19.7	30.9	
A2G2F1	976.0588 (3+)	20.6	31.3	44.0

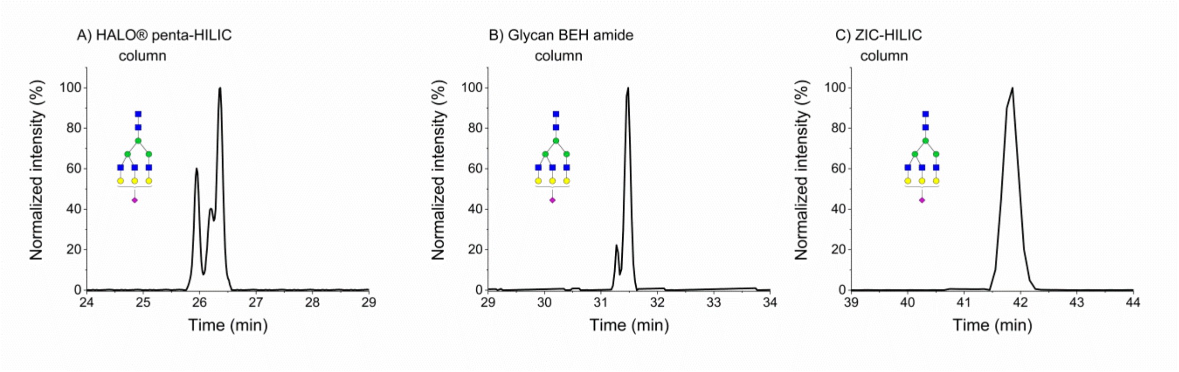

Figure S2 Normalized EIC chromatograms of A3G3S1 glycoform of the SWPAVGN187CSSALR peptide of hemopexin in HALO® penta-HILIC (A), Glycan BEH amide (B) and ZIC-HILIC (C) column.

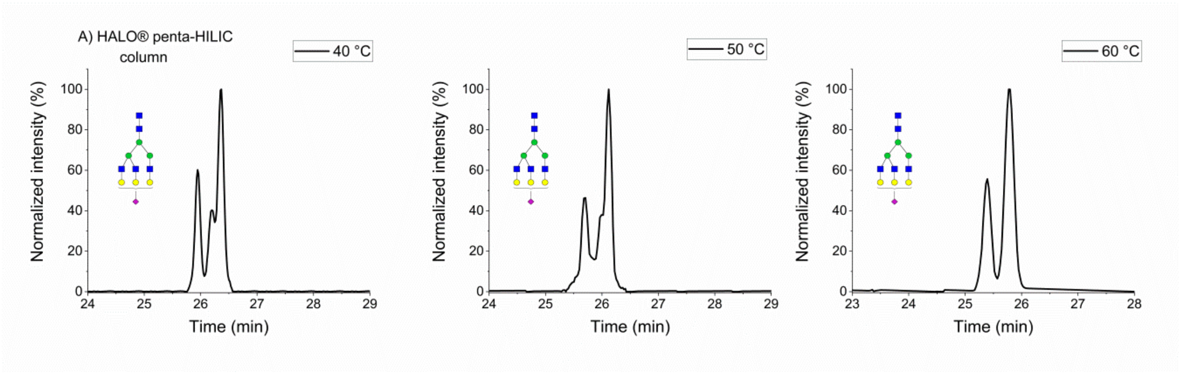

Figure S3 Separation of A3G3S1 glycoform of the of the SWPAVGN^{187}CSSALR peptide of hemopexin in HALO® penta-HILIC at different column temperatures.

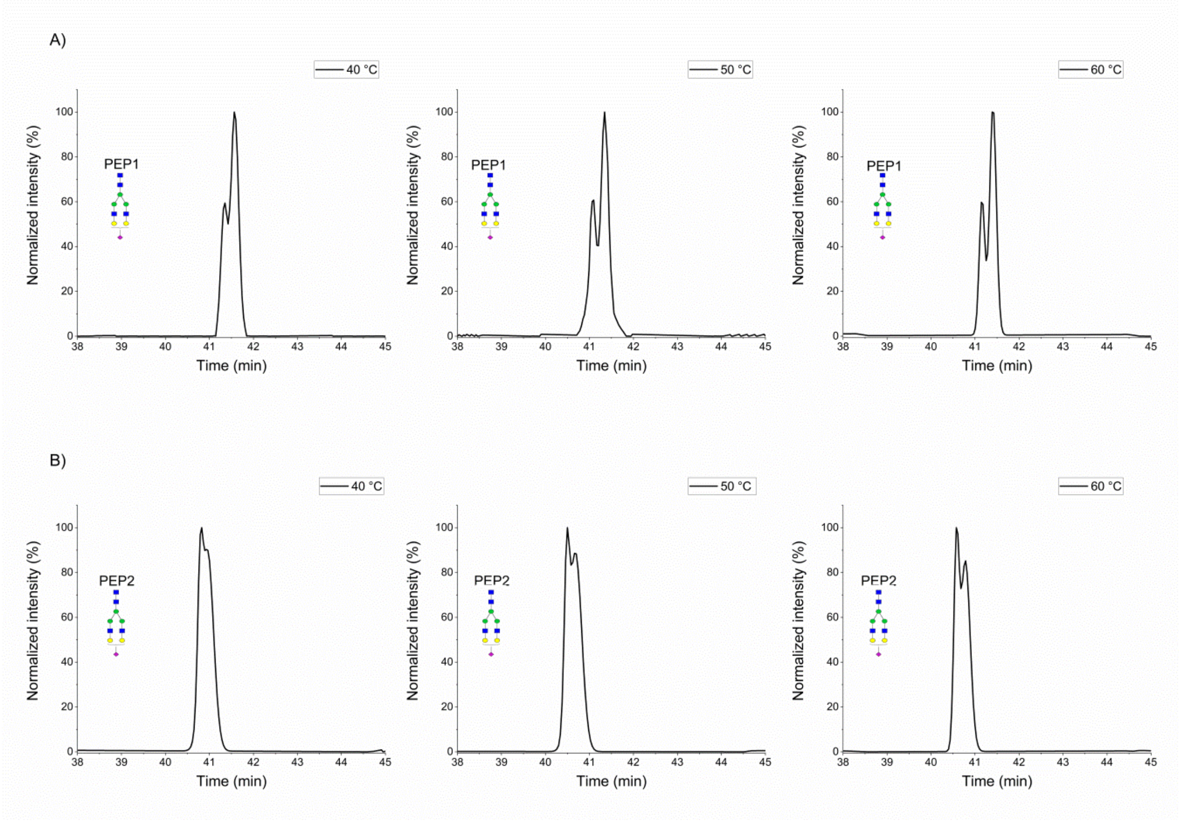

Figure S4 Separation of A2G2S1 glycoform of the of the SWPAVGN^{187}CSSALR (A) and ALPQPQN^{453}VTSLLGCTH (B) peptide of hemopexin in ZIC-HILIC column at different column temperatures. PEP1 refers to SWPAVGN^{187}CSSALR and PEP2 to ALPQPQN^{453}VTSLLGCTH.
